# Importing rescue dogs into the UK: reasons, methods and welfare considerations

**DOI:** 10.1136/vr.105380

**Published:** 2020-01-13

**Authors:** Charlotte Norman, Jenny Stavisky, Carri Westgarth

**Affiliations:** 1 School of Veterinary Science, University of Liverpool, Neston, UK; 2 Centre for Evidence-based Veterinary Medicine, University of Nottingham School of Veterinary Medicine and Science, Nottingham, UK; 3 Institute of Infection and Global Health, University of Liverpool, Neston, UK

**Keywords:** dogs, problem behaviour, surveys and questionnaires, social media, rescue, disease control

## Abstract

**Background:**

Rescuing dogs from overseas is increasing in popularity but has associated risks. This study is the first to investigate the reasons why people bring rescue dogs into the UK from overseas, the importation process, and potential welfare problems associated with this practice.

**Methods:**

An online questionnaire was advertised on social media in 2017 and received 3080 responses.

**Results:**

Participants primarily chose to adopt from abroad based on a desire for a particular dog they had seen advertised and on concern for its situation. However, some were motivated by previously having been refused dogs from UK rescues. Adopters reported that the EU Pet Travel Scheme was used to import 89 per cent of dogs, with only 1.2 per cent reportedly under the more stringent (and correct) Balai Directive. 14.8 per cent (79/533) of dogs reportedly tested for *Leishmania infantum* had positive results. Although sometimes severe, the prevalence of behavioural problems appeared comparable to that of other rescue dogs.

**Conclusion:**

It is important that vets consider testing for exotic diseases, and the provision of behavioural support, when seeing imported patients. Our findings emphasise the importance of clear guidelines on travel laws, and stricter checks on animals imported as rescues, to ensure protection against the importation of diseases that pose a risk to animal and human health in the UK.

## Introduction

The dog population of the UK is estimated at 8.5 million.^1^ Assuming a mean lifespan of 10 years (the latest research suggests it is possibly 11–12 years),^2 3^ this suggests a demand for around 850,000 dogs annually. Despite demand for dogs, approximately 10 per cent of dogs in rescue centres are euthanased annually in the UK^4^; these dogs do not appear to fit the requirements of potential owners. Interestingly, over recent years there has also been an increase in both the legal and illegal importation of dogs from outside the UK (including puppies^5^ and often involving dogs rescued from southern or eastern Europe), creating concern within the veterinary community.^6^

Campaigns by animal welfare organisations urge people to adopt, rather than purchase, a pet from a breeder. Adopting a rescue animal is a choice based on ethical/moral reasoning, but can be constrained when potential adopters are excluded based on judgements about whether they are likely to provide a good enough home.^7^ Another barrier might be the availability of the type of dog the adopter wants; in a study of factors affecting adopters’ decisions to choose a particular dog, the single most important reason was appearance.^8^ Potential adopters are willing to drive considerable distances to get the dog of their choice, and prefer having a variety from which to select.^9^ Similarly, it appears that potential adopters are also willing for the dog to travel considerable distances, but to date there has been no research surrounding the adoption of rescue dogs from abroad and their importation into the UK.

Dogs can currently be brought into the UK under two European Union (EU) regulations. The most widely recognised regulation is the EU Pet Travel Scheme (PETS; EU Regulation No 576/2013), which is for the non-commercial movement of animals whilst accompanied by their owners. An estimated 300,000 dogs enter the UK under this scheme each year, yet it is suspected that many of these are commercial imports rather than true pets.^10 11^ Dogs imported for commercial reasons, including those involving a change of ownership (such as rescue animals), should be imported under the Balai Directive (EU Regulation 92/65/ECC) and should therefore have an Intra Trade Animal Health Certificate.^12^ In 2017, around 31,000 dogs were imported in this way.^10^

Behavioural problems are an important factor in the success of adoptions, and are cited as the most common reason for the relinquishment of dogs.^13 14^ Currently there is no literature available on the behaviour of imported rescue dogs, or about how they adjust to living in the UK. It is plausible that the change in their environment and way of life is more dramatic than for a dog adopted from a rescue centre in the UK.

The importation of rescue dogs from one country to another presents an additional challenge, namely concerns about disease (including Rabies, *Echinococcus multilocularis*, Leishmaniasis, Babesiosis, Ehrlichiosis, Heptazoonosis and heartworm).^15^ The changing distribution of tick species across Europe has been linked with climate change and with increased levels of pet travel and dog importation; surveillance has found *Rhipicephalus sanguineus* and *Dermacentor variabilis* on dogs in the UK with a recent travel history.^16^ These two tick species are of concern as they are responsible for the transmission of Babesiosis and Ehrlichiosis.^17 18^ Canine babesiosis currently presents a low risk of sporadic cases throughout the UK, most likely associated with overseas travel; however, there is a new area of associated risk within the Chelmsford area, highlighting the potential for pathogen emergence within new populations.^19^ Several cases of Leishmaniasis have been confirmed in imported rescue dogs^20^ and have also resulted in transmission to another household dog with no travel history.^21^ *Linguatua serrata* has been reported in stray dogs imported from Romania, after having only previously been seen in foxes in the UK.^22^ It is currently unclear whether other cases throughout Europe reflect an expansion of core endemic areas or if they are isolated cases.^23^

Risk assessments have identified a continued requirement for Praziquantel treatment of dogs before entry to the UK, to prevent *Echinococcus multilocularis*.^24^ This requirement is specified in both the EU Pet Travel Scheme and the Balai Directive. Similarly, rabies modelling concluded that, if compliance is less than 100 per cent, the current importation laws present a higher risk than previous quarantine.^25^ Therefore, it is important to understand the common practices and levels of compliance with this treatment when importing dogs from overseas.

Clearly, the increasing phenomenon of sourcing from overseas adds further challenges when adopting a rescue dog. The aim of this study was to ascertain why people choose to adopt rescue dogs from outside the UK, and to investigate reports of health and behaviour problems with such dogs.

## Materials and methods

### Questionnaire

The data were collected using the online research software Qualtrics, through an anonymous single-use link. The questionnaire contained 44 questions (largely closed but with some open questions), which was piloted by a group of overseas-rescue dog owners and experts in this area, and refined before use. Questions related to the demographics of participants, the adoption process, the dog’s signalment, its health (including diseases tested for) and behaviour (which was based on the Behavioural Assessment for Re-homing K9s (B.A.R.K) for dogs).^26^ The survey was distributed online, predominantly by targeting Facebook groups focused on rescuing dogs from abroad, and ran for 21 days from July 26, 2017 to August 15, 2017. Participants were asked to complete the questionnaire for one dog only; if they had more than one dog, they were instructed to use the dog whose name was closest to the beginning of the alphabet.

### Participant eligibility

The first three questions were used to confirm that participants were over the age of 18 years, currently lived in the UK, and had adopted a rescue dog from outside the UK within the last five years.

### Data analysis

Data were cleaned and the statistical software Minitab was used for basic descriptive analysis. Pearson Chi-squared tests were used to assess relationships between categorical data. The qualitative research software NVIVO was used to categorise common responses to open-ended questions into codes, from which numbers of respondents were counted and illustrative quotes could be selected for presentation. Due to time constraints, a random sample of 100 responses was coded for each open question, except for the final question (which asked for any comments participants wished to make, and whether they recommended adopting from abroad); for the latter, we coded 100 responses but also presented a selection of quotes from the entire sample (selected for their ability to illustrate positive and negative experiences).

## Results

### Survey completion

Out of 3826 attempts, 3080 questionnaires were, mostly, complete and eligible, and so were used for analysis.

### Participant demographics

The majority of participants were female (93 per cent, n=2793) and married (59 per cent, n=1758). Fifty-nine per cent (n=1811) had a higher education qualification. Participants’ households were mostly families (42 per cent, n=1265) or couples (41 per cent, n=1229). A quarter of participants (24 per cent, n=716) had children younger than 16-years-old present in the household. Most participants had at least one other dog (n=2000, 67.3 per cent), some had cats (n=1093, 36.8 per cent) and 17.6 per cent (n=523) of participants had no other animals. The maximum number of dogs owned was 24. Most participants left their dogs in the house without human company for less than four hours daily (78 per cent, n=2388); few left their dogs for over eight hours (0.4 per cent, n=13).

### Dog demographics

Predominantly, the dogs imported were unknown cross-breeds (65 per cent, n=2007), but some were known crosses (17 per cent, n=516) or pure breeds (16 per cent, n=506). Eighty-one different breeds were listed, the most common of which were Pointers (22 per cent, n=104) and Podencos (10 per cent, n=47). The majority were adopted when younger than one-year old (30 per cent, n=926) or when 1–2 years old (29 per cent, n=886) and had been owned for 1–2 years (27 per cent, n=816). Female neutered dogs were most common (49 per cent, n=1500), closely followed by male neutered dogs (42 per cent, n=1288); of those neutered, 26 per cent (n=668) were neutered only after importation. Female-entire and male-entire dogs accounted for 4 per cent (n=120) and 3 per cent (n=103), respectively.

The dogs were imported from 44 countries, mainly Romania (34 per cent, n=1035), Cyprus (22 per cent, n=660) and Spain (19 per cent, n=571). Principally, dogs were put up for adoption after being found on the street (61 per cent, n=1865), whilst others were rescued from animal cruelty (10 per cent, n=299), given to a shelter by their previous owners (8 per cent, n=250) or born in a dog shelter (4 per cent, n=115), and a small number were from the dog-meat trade (1 per cent, n=21). Most participants still had their dogs (97 per cent, n=2926). Those that had been re-homed (1 per cent, n=29) went back to the originating organisation (61 per cent, n=17), eight went to friends, family or members of the public (29 per cent), three went to a UK organisation (11 per cent), and for one the destination was not reported.

### The adoption process

The majority of dogs were reportedly imported under the EU Pet Travel Scheme (89 per cent, n=2726), whereas only 37 dogs were reportedly imported under the Balai Directive (1 per cent); 260 participants did not know how their dog was imported (8 per cent). Participants predominantly adopted through an organisation (92 per cent, n=2773): 40 per cent of these were based abroad and exported dogs to the UK (n=1103), 36 per cent were UK organisations that only re-homed imported dogs (n=978), and 24 per cent were UK organisations that re-homed both imported dogs and UK dogs (n=659). Organisations based abroad were significantly more likely to export to the UK through the correct law (67 per cent) than those based in the UK (18 per cent) (P<0.0001).

The rescue organisations were the primary sources used by participants to obtain information regarding moving dogs between countries, but the GOV. UK website was also highly used (table 1). Predominantly, participants felt they had enough advice and support (90 per cent, n=2762), but 8 per cent felt they needed more (n=760). Only 37 dogs were imported into the UK under the correct Balai Directive, yet 618 participants used information from the GOV. UK website and 177 participants contacted transport companies. Interestingly, three participants rang the Department of the Environment, Fisheries and Rural Affairs (DEFRA) and one rang Heathrow Animal Reception Centre, and all four of these participants imported under the EU Pet Travel Scheme.

**Table 1:** Adoption sourcing methods reported

Finding	N	per cent*
How participants found out about the organisation	(2773)	
Social media	1475	53
Word-of-mouth	631	23
Search engine	423	15
Charity website	281	10
Other (includes meeting organisation when abroad, newspaper/magazine appeal)	355	-
How participants found the dog when not importing through an organisation	(257)	
Social media	122	45
Word-of-mouth	52	19
Whilst on holiday	49	18
Whilst living abroad	23	8
Website	22	8
Other	5	2
Sources of information used regarding moving dogs between countries		
Information from the rescue	1554	50
GOV.UK website	618	20
Social media	570	19
Friends	430	14
Airlines/Ferries/Transport	177	6
None	99	3
Other online sources	43	1
Veterinary advice	32	1
Prior knowledge	36	1
NA	3	<1
Other (eg, phone calls to DEFRA, foster person, other rescue charities or other informant)	29	-

*Do not sum up to 100% as multiple choices could be made.

**Figure 1:**
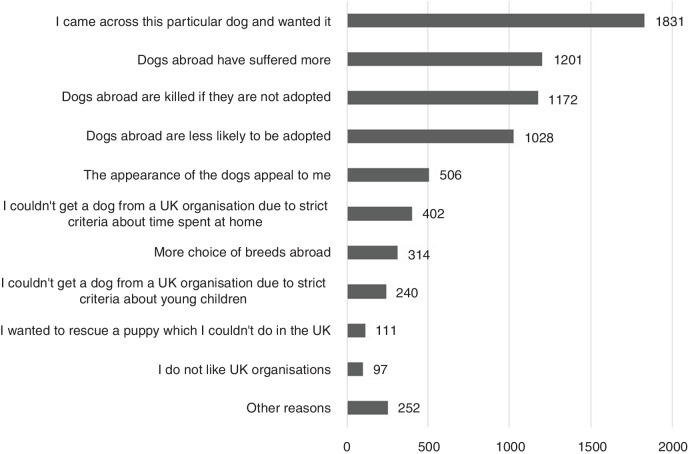
Reported reasons why participants adopted dogs from abroad.

By far the most common way of finding the dog, whether through an organisation or not, was via social media, followed by word-of-mouth (table 1). Participants were asked why they chose to adopt from abroad (figure 1), to which the primary response was ‘that they came across a particular dog and wanted it’ (n=1831, 59 per cent):

‘It’s like computer dating on Facebook. You see a dog – you read their story – you fall in love’

The next most important reasons for adoption were: perceptions of increased suffering in dogs from abroad compared to the UK (n=1201, 39 per cent) and risk of the dog being killed (n=1172, 38 per cent). The dog’s appealing appearance was another important factor (n=506, 16 per cent). Most participants had either previously adopted from rescues in the UK (53 per cent, n=1618) or, of those who had not, 39 per cent had considered adopting from the UK (n=1191). In the open responses, participants highlighted that they had tried adopting from the UK but had either not been considered suitable or the dogs available were not suitable for them:

‘When I set about adopting a dog I very nearly gave up and bought another puppy. Most of the rescues I contacted didn't return calls and those that did simply said they had nothing suitable, and others wouldn't consider me because I didn't have a fenced garden.’

‘Rules are too strict. ie, work full-time. Also, unable to find a dog I liked the look of.’

‘Dogs in UK rescues often needed adult-only home or be an only dog in household.’

Many found the process of adoption from abroad extremely easy (65 per cent, n=2003) and very few found it extremely difficult (0.5 per cent, n=14). Most participants had a home visit before adoption (n=2466, 81 per cent), 40 per cent (1201) had a lifestyle questionnaire, and 578 (19 per cent) had a phone or video interview. Few participants had no form of check (n=179, 6 per cent). The majority of dogs were specifically imported for the participant (70 per cent, n=2097), but some were already in the UK when the participant decided to adopt the dog (30 per cent, n=913).

The most common fee participants paid towards the adoption was £201–£400 (59 per cent, n=1801) and 26 per cent paid £200 or less. Only 2 per cent of participants paid over £1000 (n=58). Participants spent more if the dog was specifically imported for them (P<0.0001). Five per cent (n=156) of participants had considered returning or re-homing their dog. Participants were more likely to consider returning their dog if it was already in the UK when they adopted it (8 per cent) than if it had been imported for them (4 per cent) (P<0.0001).

### Dog behaviour

**Figure 2:**
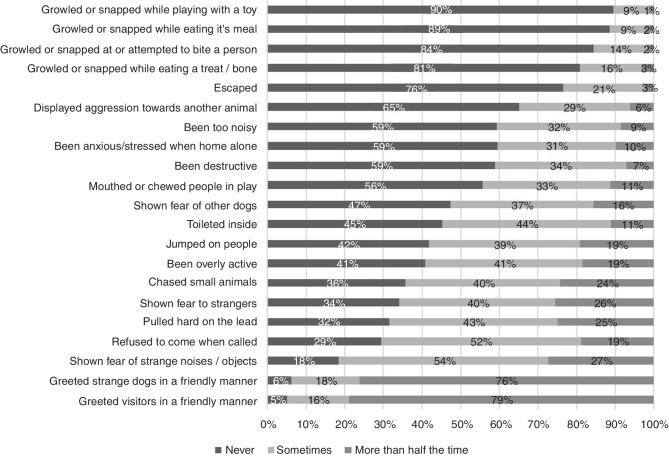
Behaviour of imported rescue dogs.

Forty-eight per cent (n=1478) of participants thought that their dog had had a behavioural test carried out before importation. Figure 2 demonstrates the reporting of different behaviours since having the dog. Common problems experienced included a fear of strange noises/objects, recall, pulling on the lead, and fear of strangers. Most participants had sought some form of training/behavioural help since adopting their dog (67.5 per cent, n=2034). Sources of advice included the internet (25 per cent, n=754), advice from friends/family (20 per cent, n=611), a private session with a dog trainer (19 per cent, n=560), advice from a vet or vet nurse (17 per cent, n=502), and a session with a behaviour counsellor (10 per cent, n=312). Fewer than one per cent of participants took advice from the organisation or person the dog was from (n=14). Of those participants who had sought training/behavioural help, 71 per cent (n=1361) said this help had resolved behavioural issues. Of those who had re-homed their dog since adoption, 20 did so due to the dog’s behavioural problems (61 per cent) and of those who had considered re-homing, 73 per cent were due to the dog’s behavioural problems.

### Dog health

The majority of dogs (85 per cent, n=2631) were up to date with UK vaccines (ie, had been vaccinated in the last year), whilst 7 per cent (n=219) were not, 0.3 per cent of owners did not know, 3 per cent stated ‘not applicable’ and 2.3 per cent stated ‘other’. Of the 72 ‘other’ responses, 40 per cent noted that their dogs were undergoing titre testing (n=27), 38 per cent of dogs were not up to date with their Leptospirosis vaccine only (n=26), 7 per cent were omitted for health reasons (n=5), 6 per cent of participants stated that they believed vaccinations were unnecessary (n=4), 4 per cent had fallen behind (n=3) and 4 per cent were using homeopathic/natural remedies (n=3).

The majority of participants believed that their dog had received, prior to importation, a full veterinary health check (93.4 per cent, n=2810), worming treatments (94.8 per cent, n=2851), flea treatments (91.4 per cent, n=2742), a blood test to confirm rabies-free status (86.8 per cent, n=2614), and tick treatments (84 per cent, n=2512). Twenty per cent (n=603) said their dog was imported to the UK with known health conditions; when specified (569), the most common of these was traumatic injury (19 per cent, n=108). Of the 20 per cent of dogs reported with healthcare issues, Leishmaniasis was the fourth most common imported health condition (9.1 per cent, n=52). Participants were asked if their dog had been tested for infectious diseases (including *Leishmania infantum, Babesia canis, Dirofilaria immitus, Ehrlichia canis, Echinococcus multilocularis*, Rabies and *Linguatula serrata)* since being in the UK (table 2). Leishmania was reported in 14.8 per cent of those tested. However, participants also showed uncertainty regarding knowledge of the tests performed; many selected ‘unknown’ in response to whether the dog was tested for Leishmaniasis (38.7 per cent), Ehrlichiosis (46.4 per cent), *Dirofilaria immitis* (50 per cent) or Babesiosis (59.4 per cent). Most participants (74 per cent, n=2267) believed they were aware of the differences in canine diseases present in the country their dog was from, compared to the UK, whilst 24.7 per cent (n=760) were unaware and 1.7 per cent (n=53) gave no answer.

**Table 2:** Owner-reported testing results for infectious diseases in imported rescue dogs

	Tested positive	Tested negative	Total tested	Apparent prevalence
Leishmaniasis (Leishmania infantum)	79	454	533	14.8%
Babesiosis/Piroplasmosis (Babesia canis)	4	301	305	1.3%
Heartworm (Dirofilaria immitis)	12	384	396	3.0%
Ehrlichiosis (Ehrlichia canis)	21	348	369	5.7%
Echinococcus multilocularis	3	288	291	1.0%
Rabies	5	529	534	0.9%
Tongueworm (Linguatula Serrata)	5	247	252	2.0%

### Other information

Participants were asked an open question regarding anything further they wished to share, including whether they would recommend adopting from abroad. Both positive and negative comments were volunteered about their experiences (table 3); however, participants often raised significant concerns about choosing reputable organisations:

**Table 3:** Examples of quotes given about the experience of adopting a rescue dog from abroad, from an open question

Positive perceptions	Negative perceptions
‘Overseas rescuers stay in touch, become friends, want to see you and the dogs happy. It's a personal experience and as adopters you are made to feel appreciated and part of a great big rescuing family.’	‘It was with us one night, jumped a 7-foot-high wall and escaped. Took 2 days to find it and capture it. We were conned.’
‘Dog has bronze and silver canine good citizen awards.’	‘Believe false passport.’
‘Dog now therapy dog.’	‘Vet estimated age 9 months. Passport says 6 years.’
‘The organisation lets you foster them until you decide it is the right dog and are ready to adopt.’	‘It was both financially and emotionally draining.’
‘My Romanian rescue is absolutely gorgeous. It’s like she knows she has been given a second chance and she is so loving and such a good girl.’	‘I was conned. The dog has epilepsy, was told we could get his pills for 5 Euros from Spain. Not the case at all.’
‘A truly rewarding experience and have made many new friends through this process. Would do it all again.’	‘I was told by them that he had a full behaviour assessment and he was great with children, other animals and people, but is slightly scared of men. In reality, he is terrified of all strangers … he acts more like a fox than a domestic dog.’
‘Definitely recommend as the rescue have supported me throughout the last 3 years of having dogs from them. Any questions they always answer and I know I can call them at any time for advice.’	‘Hard to make contact with the agency to ask questions but I guess they are busy. Alarmed that there are no follow up calls or contact of any sort after money is paid.’
‘I could choose the dog from the comfort of my own home…When out walking and talking to fellow dog walkers, most have no idea that this can all be done online via Facebook, PayPal and email. The dogs are even delivered to the door.’	‘I would say NEVER adopt a dog from abroad. Like us you can bring in diseases that shouldn't be here. There are many dogs here that need homes and having a dog with active Leishmania is no fun at all.’

Example quotes volunteered by respondents and selected by the researchers to illustrate the typical and interesting submissions. In addition, a random sample of 100 responses were coded and quantified (due to time constraints), of which 40% of respondents recommended adopting from abroad. 23% stated they would recommend the organisation that they used and 14% expressed that they were happy with the ongoing support provided by the organisation. 22% expressed that their dog had challenging behaviours, yet 25% said their dog had settled well. 12% recommended that anyone considering adopting from abroad should do proper research; similarly 7% said ‘ensure you have experience’ and 7% said ‘have realistic expectations, in particular concerning behavioural work needed’.

‘There are many that are not fully registered as charities and rip people off and use transporters that will bring rescue dogs in illegally. Some dogs are never tested for medical diseases, some arrive with distemper and heart worm. Medications are sent over with the dogs. But sometimes the dogs should not even be travelling. To cut cost, some rescues will not send dogs via the Balai rules on TRACES [Trade Control and Expert System]. Some rescues falsify passports and other documents.’

## Discussion

This study set out to investigate the reasons why people choose to rescue from abroad, the process they used to get their dog and the potential welfare implications of this practice. Participants chose to adopt from abroad due to a desire for a particular dog they came across on social media, sometimes because they felt UK rescue dogs were not suitable for their needs, or sometimes because they had been prevented from adopting from UK rescues. They perceived that dogs from abroad have undergone more suffering, are more likely to be killed and less likely to be adopted than UK rescue dogs. When they arrive, behavioural problems may be a challenge and require support. Many dogs are being imported with known health conditions and, at least to the owners’ knowledge, testing and treatment of infectious disease is not always carried out before importation. The most surprising finding of the study was that, at least according to the owners, the majority of imported rescue dogs are coming into the UK under the EU Pet Travel Scheme (EU Regulation No 576/2013), which only requires the dog to be microchipped, vaccinated for rabies and treated by a vet against *Echinococcus multilocularis*. Although this law would be appropriate in the few cases where dogs were adopted whilst the owners were on holiday or living abroad, this was inappropriate for the majority of participants. These results could partly be explained due to erroneous participant knowledge; as all dogs will have been issued a passport for transport, some participants may have been confused and assumed the dog was thus imported under the EU Pet Travel Scheme. However, this is unlikely to completely account for such stark findings. It is plausible that it could actually be occurring because PETS importation is relatively simple and cheap, whereas under the Balai Directive, dogs require a microchip, rabies vaccine, veterinary health check 48 hours before dispatch and an Intra-Trade Animal Health Certificate (ITAHC) completed by an Official Veterinarian. Additionally, the dog must come from registered or approved premises.^27^

The suggested finding of incorrect importations occurred despite the relatively large number of participants who reported seeking information on government websites regarding bringing a dog into the UK. These results indicate a lack of understanding (or application) of the laws relating to the importation of rescue dogs from abroad. Organisations based abroad were more likely to use the Balai Directive compared to organisations based in the UK, suggesting that the correct information is available and perhaps rescues based abroad are better at informing their adopters of the procedures used. Research should be conducted with importing organisations to clarify which import mechanisms are being used and to potentially educate them as to the correct importation process.

The reasons for adoption of overseas dogs were slightly different to those primarily considered in other rescue situations, but the type of dog and its appearance remained important.^8^ Breed availability in UK rescues was a barrier (for example, an oversupply of bull breeds). There was also a desire to alleviate perceived greater suffering in overseas dogs, highlighting how adopters are motivated by moral and ethical reasons.^7^ Participants predominantly found their dogs and adopted them through organisations found on social media, making it likely that they are acting on emotive images or stories they have been presented with; the rise of social media may have played a big part in the increase in adoption from abroad. The contrasting perceptions of suffering caused by differences in marketing by overseas organisations compared to UK organisations (eg, many UK rescue organisations advertise ‘no-kill’ reputations) may have inadvertently caused people to think that UK animals are less deserving. There may be lessons to be learned about the effective use of marketing and social media in encouraging adoption.

It is of note that all of our participants decided to adopt from abroad, with some specifically stating that they did so as to not to fund excess breeding. Strict requirements to adopt from UK rescue organisations, including time spent at home, the presence of young children, or criteria relating to an adopter’s house or garden, were highlighted as barriers to adopting from the UK, and this has also been reported in the USA.^7^ This raises concerns that effective ‘good’ homes might be being missed due to excessively stringent requirements and blanket policies, meaning that animals are euthanased or remain in kennels for extended periods unnecessarily. A further implication is that the requirements of overseas adoption organisations may be too relaxed, or that it is at least easier to negate the checks. An ethical but practical balance is required. Given societal changes, where pet-sitters/dog walkers are now common, people often work flexibly and at home, and most households do not have a person who does not work, a review of organisational adoption processes may be required in order to support the greater adoption of UK dogs, and so enable more people to benefit from dog ownership.

Participants that had either re-homed their dog since adoption or had considered it, most commonly did so due to behavioural problems, as previously observed.^13^ Our participants were more likely to consider re-homing their dog if it was already in the UK when it was adopted; it may be viewed as easier to return a dog if it was adopted from the UK. Although some of the behavioural problems reported for overseas dogs were severe, overall the prevalence seems comparable to UK rescue dogs. Participants reported that aggressive behaviours, including growling and snapping, were rarely expressed by their dogs and aggression was seen primarily towards other animals or when eating a treat or bone. Fearful behaviours were reported most commonly, similar to the situation with other rescue dogs.^28^ A large number of participants sought help and this resolved many behavioural issues. Some participants also commented that they were encouraged by the organisation to undertake training to help the dogs settle into their new environment. This encouragement from organisations, and the knowledge of the dog coming from another country and often from the street, may have given owners a more realistic expectation of the amount of work required to help the dog adjust, which is known to increase the likelihood of a successful adoption.^13^ Participants primarily felt that they had enough advice and support when adopting, which is a credit to the organisations and may, in particular, be related to Facebook groups linked to the rescue organisations (which were mentioned and praised by participants).

A large proportion of dogs were imported with known health conditions: many had traumatic injuries and some had Leishmania, Ehrlichia or external parasitic infections (which are a potential risk for the UK dog population). Leishmania has already been confirmed in imported rescue dogs^20^; our study has calculated an apparent prevalence (within the dogs reported in this population who have been tested for it) of 14.8 per cent. Five participants also stated that their dogs tested positive for Rabies; considering these dogs are still with the owner and rabies is tested post-mortem, this creates doubt about the knowledge of participants in relation to disease matters. Further, the tested diseases may have varied depending on what is considered to be prevalent in each country of origin, and there may have been clinical reasons to conduct tests for specific diseases. However, the health and disease status of imported dogs is a concern for the UK, and more research is required to accurately assess the risk posed by imported, infected dogs.

A concern raised by some participants was the honesty and transparency of organisations. Several participants reported that the dog’s age was vastly different to what they were originally told, some reported that the behaviour of the dog was considerably worse than explained, some reported not knowing the dog was from abroad until they had adopted it, and some believed the pet passport was fake. This raises concerns about a minority of organisations who import into the UK and who may be using sub-standard protocols or knowingly deceiving potential adopters.

A limitation to this study is the use of social media for distribution of the questionnaire; however, social media also appears to be involved in many overseas adoptions. Participants were primarily upper- or middle- class females, which echoes other research on animal adopters.^7^ The greatest limitation of this study is its reliance on owner-reported knowledge of importation practices and infectious disease testing. There may also be response bias towards those with particularly good or bad experiences. However, this study is novel, it had a large number of participants, and it provides the first research evidence into overseas-rescue dog adoption practices. Further research should be conducted to confirm importation protocols (in particular, to confirm which law is used for importation and what disease tests and treatments are performed).

In conclusion, participants adopt rescue dogs from abroad primarily due to a desire for a particular dog, usually found online. The dogs are commonly stray dogs taken from the streets in European countries, which seem to adapt surprisingly well to life in the UK. Importation appears to be through the EU Pet Passport Scheme, rather than the Balai Directive, indicating the need to give more guidance to importers about importation procedures and the use of more stringent checks. The dogs were commonly imported with known health conditions, including infectious diseases. These should be monitored pre-adoption and post-adoption, and checks during the importation process made more stringent, to ensure that UK dogs are not put at risk from exotic diseases. Potential adopters should be encouraged to gain an understanding of health and behaviour implications before adoption, and given sufficient support post-adoption. Vets also require guidance as to what to consider when presented with an overseas dog.
